# Combining prostate health index and multiparametric magnetic resonance imaging in estimating the histological diameter of prostate cancer

**DOI:** 10.1186/s12894-021-00928-y

**Published:** 2021-11-20

**Authors:** Po-Fan Hsieh, Tzung-Ruei Li, Wei-Ching Lin, Han Chang, Chi-Ping Huang, Chao-Hsiang Chang, Chi-Rei Yang, Chin-Chung Yeh, Wen-Chin Huang, Hsi-Chin Wu

**Affiliations:** 1 Department of Urology, China Medical University Hospital, No. 2, Yu-Der Rd, Taichung, 40447 Taiwan; 2 School of Medicine, China Medical University, Taichung, 40402 Taiwan; 3 Graduate Institute of Biomedical Sciences, School of Medicine, China Medical University, Taichung, 40402 Taiwan; 4 Department of Radiology, China Medical University Hospital, Taichung, 40447 Taiwan; 5 Department of Pathology, China Medical University Hospital, Taichung, 40447 Taiwan; 6 Department of Urology, China Medical University Beigang Hospital, Beigang, Yunlin 651012 Taiwan

**Keywords:** Multiparametric magnetic resonance imaging, Prostate cancer, Prostate health index, Tumor diameter

## Abstract

**Background:**

Although multiparametric magnetic resonance imaging (mpMRI) is widely used to assess the volume of prostate cancer, it often underestimates the histological tumor boundary. The aim of this study was to evaluate the feasibility of combining prostate health index (PHI) and mpMRI to estimate the histological tumor diameter and determine the safety margin during treatment of prostate cancer.

**Methods:**

We retrospectively enrolled 72 prostate cancer patients who underwent radical prostatectomy and had received PHI tests and mpMRI before surgery. We compared the discrepancy between histological and radiological tumor diameter stratified by Prostate Imaging-Reporting and Data System (PI-RADS) score, and then assessed the influence of PHI on the discrepancy between low PI-RADS (2 or 3) and high PI-RADS (4 or 5) groups.

**Results:**

The mean radiological and histological tumor diameters were 1.60 cm and 2.13 cm, respectively. The median discrepancy between radiological and histological tumor diameter of PI-RADS 4 or 5 lesions was significantly greater than that of PI-RADS 2 or 3 lesions (0.50 cm, IQR (0.00–0.90) vs. 0.00 cm, IQR (−0.10–0.20), *p* = 0.02). In the low PI-RADS group, the upper limit of the discrepancy was 0.2 cm; so the safety margin could be set at 0.1 cm. In the high PI-RADS group, the upper limits of the discrepancy were 1.2, 1.6, and 2.2 cm in men with PHI < 30, 30–60, and > 60; so the safety margin could be set at 0.6, 0.8, and 1.1 cm, respectively.

**Conclusions:**

Radiological tumor diameter on mpMRI often underestimated the histological tumor diameter, especially for PI-RADS 4 or 5 lesions. Combining mpMRI and PHI may help to better estimate the histological tumor diameter.

## Introduction

Prostate cancer is the second most common cancer in men and the fifth leading cause of death worldwide [1]. With increasingly popular screening protocols and the adoption of multiparametric magnetic resonance imaging (mpMRI) in the diagnostic pathway, an increasing number of prostate cancer cases can be diagnosed at an early stage [2]. Radical prostatectomy has long been the standard of care for clinically localized prostate cancer, and focal therapy is an emerging treatment modality which may also achieve oncological control and result in lower rates of urinary incontinence and erectile dysfunction [3, 4]. The importance of the preoperative delineation of tumor boundaries cannot be underestimated, because it may help preserve neurovascular bundles during radical prostatectomy as well as determine the safety margin during focal therapy. Specifically, the purpose of determining the safety margin for focal therapy is to safely achieve the trifecta, i.e. destruction of sufficient tissue for oncological control, whilst preserving enough normal prostatic tissue to retain both continence and potency, and carefully balance these three elements.

Based on the high sensitivity and specificity for clinically significant prostate cancer, mpMRI is widely used to assess the tumor location and volume [5]. The tumor diameter on mpMRI has been correlated with extraprostatic extension, seminal vesicle invasion, and positive margin after radical prostatectomy [6]. Nevertheless, mpMRI is limited by a low positive predictive value for Prostate Imaging-Reporting and Data System (PI-RADS) 3 or 4 lesions and high inter-reader variability [7, 8]. The staging accuracy of mpMRI has also been reported to vary across risk groups [9]. Moreover, mpMRI often underestimates the histological tumor boundary [10, 11]. Although a safety margin of at least 5–10 mm has been suggested for focal therapy of prostate cancer, how to individualize the safety margin for each patient remains unknown [10, 12–14]. Recently, a pilot study suggested performing intraoperative digital analysis of ablation margins using fluorescent confocal microscopy given the relevance of MRI targeting errors and the presence of MRI invisible cancer [15]. On the other hand, prostate health index (PHI) is a serum biomarker which has a higher specificity compared with prostate specific antigen (PSA) [16]. Some studies have reported that PHI is significantly correlated with tumor volume and adverse pathological outcomes [17–22].

Our previous work showed that the combination of PHI and mpMRI had a higher accuracy for detecting clinically significant prostate cancer compared with PHI or mpMRI alone [23]. Therefore, the aim of the present study was to evaluate the feasibility of combining PHI and mpMRI to estimate the histological tumor diameter and determine the safety margin during treatment of prostate cancer.

## Materials and methods

We retrospectively collected patients with biopsy-proven prostate cancer who underwent robot-assisted laparoscopic radical prostatectomy (RALRP) from January 2016 to December 2020 and had received preoperative PHI tests and mpMRI at a tertiary referral center. The exclusion criteria were metastatic prostate cancer (N1 or M1), PHI tested during an episode of urinary tract infection, defined as pyuria (3 or more white blood cells per high power field of unspun, voided mid-stream urine), or poor quality of mpMRI, which included inadequate field of view, inadequate in-plane resolution, inadequate slice thickness, lack of multiple and high *b* values, unclear delineation of anatomical landmarks, or the presence of artifacts. This study was approved by the Research Ethics Committee of China Medical University and Hospital, Taichung, Taiwan (Protocol Number: CMUH110-REC3-048). The need for informed consent was waived by the Research Ethics Committee because all clinical practice in this study was routine work for prostate cancer patients and carried out in accordance with EAU guidelines [3]. In addition, the retrospective analysis did not influence any clinical decision making or violate the patients’ rights.

### PHI and mpMRI

PSA parameters including total PSA, free PSA, and p2PSA were tested using a Beckman Coulter DxI 800 Immunoassay System (Beckman Coulter, Taiwan Inc.) to obtain PHI (PHI = (p2PSA/free PSA) × √PSA) before prostate biopsy.

Prostate mpMRI examinations were done using a 3-T scanner (Signa HDxt, GE Healthcare, Milwaukee, WI) with a four-channel high definition (HD) cardiac array coil before prostate biopsy or at least 6 weeks after prostate biopsy. The mpMRI protocol included T2-weighted imaging (T2WI), dynamic contrast enhanced (DCE) imaging, diffusion-weighted imaging (DWI) with *b* values of 0 and 1000–1400 s/mm^2^, and apparent diffusion coefficient (ADC) mapping. All mpMRI scans were interpreted by an experienced radiologist (W. C. L.) who had 12 years of prostate MRI experience with > 500 scans per year, and reported according to PI-RADS version 2 [24]. The index lesion was defined as the single lesion with the highest PI-RADS score. If there were two or more lesions with the same PI-RADS score, the index lesion was defined as the largest one. We measured the maximal diameter of the index lesion on the axial, coronal, or sagittal view of T2WI to determine the radiological tumor diameter.

### Prostate biopsy

For men who underwent mpMRI before biopsy, we performed targeted biopsies for at least 2 cores and systematic biopsies for at least 12 cores. Initially, we performed transrectal cognitive biopsies based on lesions revealed on mpMRI. In April 2019, we introduced the MRI/ultrasound fusion platform, BioJet® (D&K Technologies GmbH, Barum, Germany), and thereafter we exclusively performed transperineal software fusion biopsies. For men who did not undergo mpMRI before biopsy, we performed systematic biopsies for at least 12 cores.

### Histology

RALRP was performed by urologists who were specialists in the procedure. The prostatectomy specimens were fixed with 10% neutral buffered formalin for 24 h, and subsequently each entire prostate specimen was sectioned. The proximal (bladder neck) margins were thinly shaved. We amputated the distal 1 cm of the apex, and then sectioned this apical cone at right angles to the cut edge in thin parallel slices to accurately assess the distal margin. After the margins had been measured, we serially sectioned the prostate at 3-mm intervals from the apex to base. Individual slices were sectioned carefully to maintain the orientation. To fit into standard tissue cassettes, a median section was made through the urethra to divide the slice into right and left sides, and a coronal section was made through the urethra to further divide the slice into anterior and posterior quadrants. All slices were performed using routine tissue processing and embedding in paraffin, and 4 μ thick slices were cut from these paraffin blocks and stained by hematoxylin and eosin (H&E). A uropathologist (H. C.) who had 22 years of experience with > 100 cases per year reviewed all of the specimens. The grading of prostate cancer was in accordance with the 2014 International Society of Urological Pathology Consensus Conference guidelines [25]. All of the cases were reviewed in a multidisciplinary team meeting by urologists, radiologists and pathologists to confirm the concordance of the index tumor on histology and mpMRI. The histological tumor diameter was defined as the maximal diameter of the index tumor on histology.

### Statistical analysis

Continuous variables were reported as median (interquartile range, IQR) or mean (standard deviation, SD) and 95% confidence interval (CI), and categorical variables were reported as proportions. The normality of data was tested using the Kolmogorov–Smirnov test. We performed Pearson correlation analysis between histological tumor diameter and clinical parameters including age, PSA, PHI, radiological tumor diameter, biopsy/histology grade group, and histology T stage. We then compared the median discrepancy between radiological and histological tumor diameter stratified by different PI-RADS score, PSA, biopsy grade group, and PHI using the Mann–Whitney U test or Kruskal–Wallis test. Finally, we separated the study population into low PI-RADS (2 or 3) and high PI-RADS (4 or 5) groups and assessed the influence of PHI on the discrepancy in each group. All statistical analyses were conducted using SPSS version 22 (IBM Corp, Armonk, NY, USA), assuming a two-sided test with an alpha of 5% for statistical significance.

## Results

Overall, 572 patients received RALRP, of whom 564 patients underwent mpMRI before surgery. Of these patients, 76 (13.5%) had PHI tests. Four patients were excluded; one had a concurrent urinary tract infection during PHI test, one had lymph node involvement on pathology, one had prostate cancer diagnosed by transurethral resection of the prostate, and one had poor quality mpMRI due to motion artifacts. Finally, a total of 72 men were enrolled in this study, and their clinical characteristics are shown in Table 1. The median age was 66 (IQR 60–69) years old. The median PSA level was 9.03 (IQR 5.95–13.36) ng/mL, and the median PHI was 54.97 (IQR 42.41–78.04). Forty-two men underwent mpMRI before the prostate biopsy, including a transrectal cognitive biopsy in 29, and a transperineal software fusion biopsy in 13. Thirty men underwent mpMRI after a systematic biopsy, and the mean duration between biopsy and mpMRI was 58.6 (SD 40.4, range 42–252) days. The PI-RADS scores of the index tumor were 2, 3, 4, and 5 in 4, 3, 29, and 36 men, respectively. The mean radiological and histological tumor diameters were 1.6 (SD 0.89, 95% CI [1.39, 1.81]) cm, and 2.13 (SD 1.11, 95% CI [1.86, 2.39]) cm, respectively. The mean discrepancy between radiological and histological tumor diameter was 0.53 (SD 0.67, 95% CI [0.37, 0.68]) cm. The Kolmogorov–Smirnov test revealed that the histological tumor diameters were normally distributed, while the radiological tumor diameters and their discrepancy were not normally distributed.

**Table 1:** Patient characteristics

Parameters	Overall population (n = 72)
Age (years), median (IQR)	66 (60–69)
PSA (ng/mL), median (IQR)	9.03 (5.95–13.36)
Free PSA/PSA (%), median (IQR)	12.76 (9.38–17.39)
PSA density (ng/mL), median (IQR)	0.26 (0.16–0.37)
%p2PSA, median (IQR)	2.04 (1.4–2.5)
PHI, median (IQR)	54.97 (42.41–78.04)
PI-RADS score, n (%)	
2	4 (5.6)
3	3 (4.2)
4	29 (40.3)
5	36 (50)
Radiological tumor diameter (cm), mean ± SD (95% CI)	1.6 ± 0.89 (1.39–1.81)
Biopsy cores, median (IQR)	
Targeted biopsy + systematic biopsy	4 (2–8) + 15 (12–18)
Systematic biopsy alone	14 (12–16)
Biopsy grade group, n (%)	
1	25 (34.7)
2	19 (26.4)
3	13 (18.1)
4	12 (16.7)
5	3 (4.2)
Histology grade group, n (%)	
1	10 (13.89)
2	28 (38.89)
3	28 (38.89)
4	1 (1.39)
5	5 (6.94)
Histology T stage, n (%)	
2	52 (72.2)
3a	16 (22.2)
3b	3 (4.2)
4	1 (1.4)
Histological tumor diameter (cm), mean ± SD (95% CI)	2.13 ± 1.11 (1.86–2.39)
Discrepancy between histological and radiological tumor diameter (cm), mean ± SD (95% CI)	0.53 ± 0.67 (0.37–0.68)

PHI: Prostate Health Index; PI-RADS: Prostate Imaging Reporting and Data System; PSA: prostate specific antigen; %p2PSA: percentage of p2PSA to free PSA

Table 2 shows the Pearson correlation analysis between the histological tumor diameter and clinical parameters. The histological tumor diameter was highly correlated with radiological tumor diameter (*r* = 0.80, *p* < 0.01). In addition, the histological tumor diameter was also correlated with PHI (*r* = 0.52, *p* < 0.01), histology grade group (*r* = 0.37, *p* < 0.01), biopsy grade group (*r* = 0.32, *p* = 0.01), histology T stage (*r* = 0.32, *p* = 0.01), and PSA (*r* = 0.30, *p* = 0.01).

**Table 2:** Pearson correlation between histological tumor diameter and clinical parameters

Parameters	*r*	*p* value
Age	0.11	0.38
PSA	0.30	0.01
Free/total PSA	−0.20	0.10
PSA density	0.22	0.06
%p2PSA	0.21	0.08
PHI	0.52	< 0.01
Radiological tumor diameter	0.80	< 0.01
Biopsy grade group	0.32	0.01
Histology grade group	0.37	< 0.01
Histology T stage	0.32	0.01

PHI: Prostate Health Index; PSA: prostate specific antigen; %p2PSA: percentage of p2PSA to free PSA

The median discrepancy between radiological and histological tumor diameter of PI-RADS 4 or 5 lesions was significantly greater than that of PI-RADS 2 or 3 lesions (0.5 cm vs. 0 cm, *p* = 0.02). On the other hand, the median discrepancy between radiological and histological tumor diameter was not significantly correlated with PSA (*p* = 0.36), biopsy grade group (*p* = 0.07), or PHI (*p* = 0.55).

**Fig. 1:**
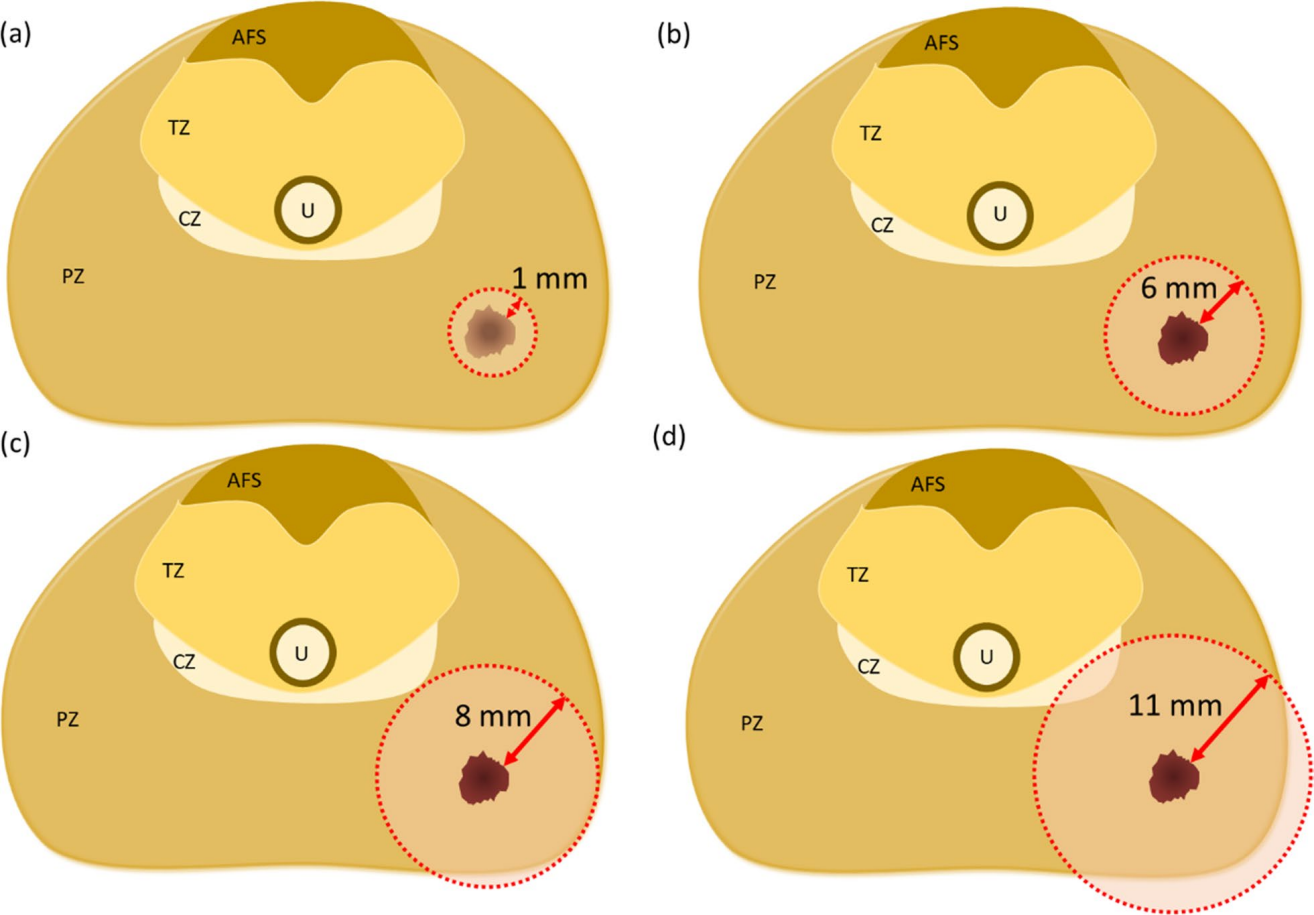
The schematic diagram the safety margin of PI-RADS 2 or 3 lesions (**a**), and safety margin of PI-RADS 4 or 5 lesions stratified by PHI < 30 (**b**), 30 ≤ PHI ≤ 60 (**c**), PHI > 60 (**d**). AFS: anterior fibromuscular stroma; CZ: central zone; PHI: Prostate Health Index; PI-RADS: Prostate Imaging Reporting and Data System; PZ: peripheral zone; TZ: transition zone; U: urethra

Finally, in the low PI-RADS group, the upper limit of the discrepancy between radiological and histological tumor diameter was 0.2 cm. In the high PI-RADS group, the upper limits of the discrepancy between radiological and histological tumor diameter were 1.2 cm, 1.6 cm, and 2.2 cm in men with PHI < 30, 30–60, and > 60, respectively. Assuming that the tumor had a spherical shape, the safety margin could be set at 0.1 cm for PI-RADS 2 or 3 lesions. For PI-RADS 4 or 5 lesions, the safety margins could be set at 0.6 cm, 0.8 cm, and 1.1 cm when the PHI was < 30, 30–60, and > 60, respectively (Fig. 1).

## Discussion

In this study, we found that the radiological tumor diameter on mpMRI underestimated the histological tumor diameter by a mean of 0.53 cm. In addition, the discrepancy between radiological and histological tumor diameter of PI-RADS 4 or 5 lesions was greater than that of PI-RADS 2 or 3 lesions. For PI-RADS 2 or 3 lesions, a safety margin of 0.1 cm may be sufficient. For PI-RADS 4 or 5 lesions, the safety margins should be 0.6 cm, 0.8 cm, and 1.1 cm when the PHI is < 30, 30–60, and > 60, respectively. To the best of our knowledge, this is the first study to estimate histological tumor diameter and safety margin using a combination of PHI and mpMRI.

Accurately estimating the tumor boundary is key to successful focal therapy, and it can also decrease the positive margin rate after radical prostatectomy. Although mpMRI is the most commonly used tool to estimate tumor volume [5], underestimation of the histological tumor volume has frequently been reported [10, 11]. This may be because at the periphery of the index tumor the cancer may be low grade or intermixed with normal prostatic tissue, making the tumor inconspicuous on mpMRI [26]. In this study, we found that mpMRI underestimated the histological tumor diameter by a mean of 0.53 cm. Consistent with Le Nobin’s study [10], the discrepancy between radiological and histological tumor diameter was greater in PI-RADS 4 or 5 lesions compared with PI-RADS 2 or 3 lesions. To achieve oncological control, a safety margin beyond the index lesion is necessary during any treatment of localized prostate cancer. However, there is currently no consensus on how to determine the safety margin. Practically, the best strategy currently available to estimate tumor volume is to combine biopsy results and MRI information. Specially directed biopsies around the lesion may assist in margin determination. In addition, several nomograms incorporating 4 K score, clinical and imaging parameters have been developed to predict the presence of cancer outside the index lesion and the added value of systematic biopsies [27, 28].

PHI is a serum biomarker for prostate cancer, and it has been associated with cancer detection, stage, grade, surgical margin status, and tumor volume [21, 22, 29]. Previous studies have reported area under the curve values of PHI to predict a tumor volume greater than 0.5cm^3^ ranging from 0.72 to 0.94 [18–20]. Friedersdorff et al. reported that PHI had a significantly higher correlation with tumor volume than Gleason score (Pearson’s *r* = 0.588 vs. 0.385, *p* = 0.008) [20]. In our study, PHI was significantly correlated with histological tumor diameter (*r* = 0.52, *p* < 0.001). The correlation coefficient of PHI was superior to that of PSA (*r* = 0.30) and biopsy grade group (*r* = 0.32), and just inferior to radiological tumor diameter (*r* = 0.80).

Importantly, we used PHI to calibrate tumor diameter on mpMRI. For PI-RADS 2 or 3 lesions, the discrepancy between radiological and histological tumor diameter was 0.2 cm, which means that extending of the radius of the index lesion by 0.1 cm may be sufficient to cover the tumor boundary. On the other hand, for PI-RADS 4 or 5 lesions, the safety margins should be extended to 0.6 cm, 0.8 cm, and 1.1 cm when the PHI is < 30, 30–60, and > 60, respectively. In other words, combining PHI and mpMRI may be able to estimate the histological tumor diameter more accurately than using mpMRI alone. Individualized treatment margins can help to decide whether neurovascular bundles can be preserved and how wide the bladder neck or urethra should be resected. It may also be possible to achieve complete tumor destruction during focal therapy, and preserve more normal prostatic parenchyma to lower the risk of injury to neurovascular bundles, external sphincter, bladder neck and urethra. Namely, the combination of imaging and serological biomarkers has the potential to achieve the best oncological outcomes whilst preserving functional outcomes including continence and sexual function. Future interventional studies on PHI and mpMRI are warranted to clarify whether these endpoints can be achieved.

In this study, we used the maximal tumor diameter as a surrogate of tumor volume. In fact, there is still no robust evidence for how best to measure tumor volume on mpMRI [30]. Tumor volume can also be estimated using planimetric maps or three-dimensional quantification, which require time for segmentation and lesion contouring [10, 31]. In contrast, measuring the maximal tumor diameter on mpMRI is a relatively simple and practically feasible method. Mizuno et al. reported that the maximal tumor diameter on mpMRI had a higher correlation with extraprostatic disease than maximal tumor area or total tumor volume [32]. In our study, the histological tumor diameter was significantly correlated with radiological tumor diameter (*r* = 0.80, *p* < 0.01), pathological T stage (*r* = 0.32, *p* = 0.01) and grade group (*r* = 0.37, *p* < 0.01). That is, the tumor diameter may help to predict cancer aggressiveness and prognosis. Research on prostate MRI radiomics and machine learning may lead to automatic lesion localization, volumetry, and assessment of tumor biology in the future [33, 34].

There are several limitations to this study. First, only 7 men were enrolled in the low PI-RADS group. This is because biopsies are not routinely suggested for PI-RADS 2 or 3 lesions unless the patient has a high clinical suspicion of prostate cancer. The limited number of cases may have reduced the power of comparisons between the high PI-RADS and low PI-RADS groups. The overall small sample size also precluded us from adding a validation cohort. However, our preliminary study addresses an important issue. Prospective large-scale studies, either in a real-world or in silico setting, are needed to assess the combined value of PHI and mpMRI in estimating tumor volume. Second, there was selection bias associated with the RALRP-only population. Other modalities of radical prostatectomy, including open or laparoscopic procedures, are needed to validate our results. Third, patients with mpMRI scheduled before or after a prostate biopsy were enrolled. Awareness of the biopsy outcomes may have affected the radiologist’s interpretation of post-biopsy mpMRI. Nevertheless, the mean duration between biopsy and mpMRI was up to 58.6 days, and the small amount of residual hemorrhage did not influence identification of the index lesion. Fourth, the prostate biopsy protocol was not uniform. Systematic biopsy alone was performed in the men without pre-biopsy mpMRI, and the lack of targeted biopsy may have resulted in undergrading of the biopsy outcome. Fifth, this study was conducted among an Asian population. Although a recent study showed similar staging accuracy of mpMRI in African Americans and Caucasian Americans, the application of combining PHI and mpMRI needs to be validated in different races [35]. Finally, we assessed the diameter of the index tumor based on the hypothesis that the index tumor drives the natural course of prostate cancer and determines the prognosis [36]. However, prostate cancer is usually a multifocal disease. The prognostic roles of satellite lesions are variable and remain to be investigated, and the combination of mpMRI, biomarkers, and genetic signatures may help in decision making for the treatment of prostate cancer [37].

## Conclusions

In conclusion, the radiological tumor diameter on prostate mpMRI often underestimated the histological tumor diameter, especially for PI-RADS 4 or 5 lesions. The combination of mpMRI and PHI may help to better estimate the boundary of prostate cancer and refine the procedures of radical prostatectomy and focal therapy. Prospective, large-scale studies are needed to validate our results.

## Data Availability

The data supporting the conclusions are contained within the manuscript. The datasets used and analyzed during the current study are available from the corresponding author on reasonable request.
